# Epidemiology of Injuries in Men’s Professional and Amateur Football (Part I)

**DOI:** 10.3390/jcm12175569

**Published:** 2023-08-26

**Authors:** Tudor Vladimir Gurau, Gabriela Gurau, Doina Carina Voinescu, Lucretia Anghel, Gelu Onose, Daniel Andrei Iordan, Constantin Munteanu, Ilie Onu, Carmina Liana Musat

**Affiliations:** 1 Faculty of Medicine and Pharmacy, ‘Dunarea de Jos’ University of Galati, 800008 Galati, Romania; gurauvlad123@gmail.com; 2 Department of Morphological and Functional Sciences, Faculty of Medicine and Pharmacy, ‘Dunarea de Jos’ University, 800008 Galati, Romania; gabigurau@yahoo.com (G.G.); carmina.musat@ugal.ro (C.L.M.); 3 ‘Sf. Apostol Andrei’ Clinical Emergency County Hospital, 800578 Galați, Romania; carinavoinescu@gmail.com (D.C.V.); anghel_lucretia@yahoo.com (L.A.); 4 Clinical Medical Department, Faculty of Medicine and Pharmacy, ‘Dunarea de Jos’ University, 800008 Galati, Romania; 5 Neuromuscular Rehabilitation Clinic Division, Teaching Emergency Hospital “Bagdasar-Ar-Seni”, 041915 Bucharest, Romania; gelu.onose@umfcd.ro; 6 Faculty of Medicine, University of Medicine and Pharmacy “Carol Davila”, 020022 Bucharest, Romania; 7 Department of Individual Sports and Kinetotherapy, Faculty of Physical Education and Sport, ‘Dunarea de Jos’ University of Galati, 800008 Galati, Romania; 8 Center of Physical Therapy and Rehabilitation, ‘Dunărea de Jos’ University of Galati, 800008 Galati, Romania; 9 Department of Biomedical Sciences, Faculty of Medical Bioengineering, University of Medicine and Pharmacy “Grigore T. Popa” Iaşi, 700454 Iaşi, Romania; constantin2378@yahoo.com

**Keywords:** accidents, football, professional, amateur, men

## Abstract

Background (1): Football is the most popular sport among men, associated with a certain risk of injury, which leads to short- and long-term health consequences. While the injury profile of professional footballers is known, little is known about the injury profile of amateur footballers; amateur football is a major and diverse area, the development of which should be a priority for football associations around the world and UEFA. The aim of this study was to perform a systematic review of epidemiological literature data on injuries in professional and amateur football players belonging to certain leagues. Methods (2): A systematic review according to the PRISMA guidelines was performed until June 2023 in the databases PubMed, Web of Science, Google Academic, Google Scholar, and Diva portal. Forty-six studies reporting injury incidence in professional and amateur men’s football were selected and analyzed. Two reviewers independently extracted data and assessed study quality using an adapted version of the Strengthening the Reporting of Observational Studies in Epidemiology (STROBE) statement and the Newcastle Ottawa Scale (NOS) to assess risk of bias for the quality of external validity. Results (3): The overall incidence of injuries in professional male football players was 7.75 ± 2.28, 95% confidence interval, injuries/1000 h of exposure and that of amateur football players was 7.98 ± 2.95, 95% confidence interval, injuries/1000 h of exposure. The incidence of match injuries (30.64 ± 10.28, 95% confidence interval, injuries/1000 exposure hours) was 7.71 times higher than the training injury incidence rate (3.97 ± 1.35, 95% confidence interval, injuries/1000 h) in professional football players and 5.45 times higher in amateurs (17.56 ± 6.15 vs. 3.22 ± 1.4, 95% confidence interval, injuries/1000 h). Aggregate lower extremity injuries had the highest prevalence in both categories of footballers, being 83.32 ± 4.85% in professional footballers and 80.4 ± 7.04% in amateur footballers: thigh, ankle, and knee injuries predominated. Conclusions (4): Professional and amateur football players are at substantial risk of injury, especially during matches that require the highest level of performance. Injury rates have implications for players, coaches, and sports medicine practitioners. Therefore, information on football injuries can help develop personalized injury risk mitigation strategies that could make football safer for both categories of football players. The current findings have implications for the management, monitoring, and design of training, competition, injury prevention, especially severe injury, and education programs for amateur football players.

## 1. Introduction

Football is a team sport played between two teams. Currently, football is played by about 270 million players from over 200 countries, which makes it the most popular sport in the world [1,2,3]. According to FIFA and sports researchers, millions of people play professional, semi-professional, or amateur football. This sport is practiced both by adults (men and women) and by young people in junior groups, which represent approximately 4% of the world’s population [4,5]. Football involves physical contact between players, high-intensity movements, rapid changes of direction, sprints, accelerations, and kicking the ball, which can cause traumatic and overuse injuries [6,7,8,9]. Professional football has one of the highest incidence rates of injuries, resulting in player unavailability for training and matches, high medical costs, reduced team performance, and personal suffering for the injured player. In fact, the risk of injury has been shown to be approximately 1000 times higher in professional football players compared to common industrial occupations, generally considered to have a high risk of accidents [10].

Injuries to adult football players during both training and matches are widely documented, although methodological inconsistencies make comparisons difficult.

The incidence, pattern, and characteristics of football injuries, such as the type and nature of the injury, anatomical sites of injury, severity, mechanisms, and biomechanical, anthropometric, and physiological aspects of football players, have been investigated and described in previous studies.

In professional football players, the incidence of injuries during matches ranged from 12 to 66.0/1000 h and from 1.5 to 7.6/1000 h of training exposure [4,11,12,13,14,15]. Most epidemiological studies have shown that the number of injuries during competition is approximately 4–6 times higher than in training. Ankle and knee sprains and hamstring and groin muscle strains are the dominant injury types and account for more than 50% of all football injuries. Over 65% of football injuries are classified as minor, 25% moderate, and 10% serious. About 50% of football injuries occur from direct contact from one player to another, including collisions and tackling, while the rest (non-contact) occur during actions such as running, shooting, and turning the head [4,16,17,18,19,20,21,22,23].

A wealth of research has focused on injuries in professional football, but millions of athletes play football at a non-professional level. Football injuries affect the quality of life of the player and constitute a serious threat to a career [24]. Expertise on injury rates in male amateur football players is limited [19,25,26,27,28,29,30]. The incidence of injuries varies widely from 4.7 to 36.9 injuries per 1000 h of match exposure and 0.9–6.0 injuries per 1000 h of training exposure [25,26]; the incidence rates vary from study to study, probably due to methodological differences and the type of sample examined.

The primary aim of our study was to conduct a systematic review of the literature quantifying the incidence of injuries in male professional and amateur football. Secondarily, we sought to highlight general effects regarding location, type, severity, mechanism of injuries, and type of incident.

## 2. Method

Our review was designed in accordance with the Guidelines of Protocols for Systemic Revisions of Observational Studies in Epidemiology, PRISMA (Preferred Reporting Items for Systematic Reviews and Meta-Analyses) [31,32,33]. The PRISMA checklist is presented in Supplementary Table S1 online.

### 2.1. Search Strategy and Eligibility Criteria

A review of a relatively large number of studies identified in the electronic databases PubMed, Medline, Google Scholar, and Diva Portal was performed up to June 2023. Keywords entered were the following: epidemiology football injuries, male football injuries, professional football, amateur football, incidence, location, type, and severity of injuries.

The search and selection of studies was performed by 2 co-authors. Disagreements between the 2 reviewers were resolved by discussion, without the need for the intervention of a third reviewer to reach a consensus. To be considered eligible for inclusion, studies had to meet some criteria.

Inclusion criteria for the selected articles considered were as follows: prospective and retrospective longitudinal research studies, original research studies, systematic reviews, and meta-analyses; studies that included professional or amateur football players (male) as participants aged 18 years or older in professional or 17 years or older in amateur football players, participating in a senior men’s football league (elite club or club by amateurs) [34] or the international senior men’s football tournament (match between two national teams); and studies that provided information on injury incidence (overall, match, and training) and/or location, type, and severity of injuries; studies that defined injury in terms of time loss and calculated the total, training, or match incidence as injuries/1000 h of exposure [16] and expressed injuries as percentages by location, type, and severity or provided sufficient data that allowed their calculation by standardized equations or percentage.

Additional criteria: studies had to be full-text articles or abstracts published in peer-reviewed journals before June 2023, published in English.

Exclusion criteria: studies that provided hypotheses, reasoning, and methodologies and not study results (protocol studies); studies that included women, children, and adolescents under 17 years of age; studies that presented incomplete data or data from a study already included; studies that used definition of injury other than time loss; and studies related to sports other than football. Literature reviews, non-conclusive abstracts, editorial comments, and letters to the editor were also excluded.

### 2.2. Data Extraction

A data extraction sheet specially created in Microsoft Excel was used to extract data from each eligible study. Data extraction was performed by two reviewers (AB and CD) independently. A third rater (EF) was consulted to resolve disagreements between these raters and facilitate consensus. The data extraction sheet included the following items: (a) general descriptors of the study, (b) characteristics of the studied population, (c) epidemiological results (lesions and their characteristics: location, type, severity, and mechanism), and (d) data on exposure [34]. Operational definitions and moderator variables are presented in Supplementary Tables S2–S4.

### 2.3. Quality and Risk of Bias Assessment

The reporting quality of included studies was assessed using an adapted version of the Strengthening the Reporting of Observational Studies in Epidemiology (STROBE) statement by [20]. Supplementary Table S5 provides a description of the 11 STROBE criteria, designed to assess the quality of studies included in the systematic review. Items on the STROBE statement were scored 0 or 1 point, awarded for each item on the checklist. Using this quality checklist, a maximum score of 11 indicates that the article meets the requirements for a high-quality publication.

In addition, an adapted version of the Newcastle Ottawa Scale (NOS) was used to assess risk of bias for the quality of external validity. The criteria proposed by [20] comprise 8 items: 1. Study setting (description type of football players, location, and period); 2. Definition of the damage; 3. The representativeness of the exposed cohort; 4. Exposure defining and measuring; 5. Demonstrating that the outcome of interest was not present at the beginning of the study; 6. Evaluation of the results; 7. There was a long enough follow-up for the results to occur; 8. Adequacy of cohort monitoring. This scale uses the star rating system to indicate the quality of a study (maximum 8 stars). The higher the number of stars given to an article, the lower the risk of bias. The evaluation of the articles included in the study was performed independently by two co-authors (AB and CD), with disagreements being resolved by consensus. Supplementary Table S6 displays a brief description of each article from the adapted version of the NOS used in systematic reviews and meta-analyses. The star was awarded only to studies who met the criteria.

### 2.4. Statistical Analyses

All statistical analyses were performed with Windows Excel 2010. Descriptive data such as percentage lesion characteristics were calculated as mean values and standard deviations (±SD). Where incidence rates were not reported but the required elements were presented, they were calculated using the following formula:

Incidence = (Number of injuries/Number of hours of general, match, or practice exposure) × 1000

Confidence intervals (95% CI) were calculated as follows [19]:

Lower bound (95% CI) = Incidence/e^1.96 × [Square root (1/Number of injuries)]^

Upper bound (95% CI) = Incidence × e^1.96 × [Square root (1/Number of injuries)]^

Correlations between overall, training, and match incidence were determined by calculating the Pearson coefficient (Correl), which indicates the relationship between two variables. The significance level was set at 0.05.

## 3. Results

### 3.1. Descriptive Characteristics of the Studies

**Figure 1:**
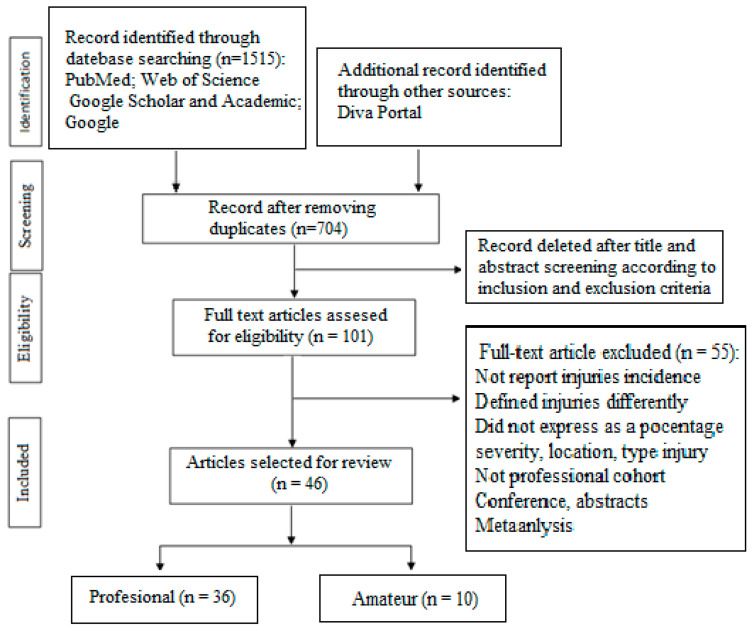
PRISMA flow chart.

A number of 1525 references were identified by using the search strategies, of which 720 were excluded at the first check as duplicates (47.2%). Of the remaining 805 (52.8%), 704 (46.2% in total) were eliminated after reading the titles and abstracts. They referred to injuries in other sports (*n* = 214; 14.0%); in children, adolescents, and young athletes under the age of 17 (*n* = 257; 15.5%); in women’s football (*n* = 161; 10.6%); and other subjects (*n* = 72; 4.7%). The remaining full-text articles and some conclusive abstracts (*n* = 101; 6.6%) were screened for eligibility, referring to adult professional and amateur football players. Of these, *n* = 55; 3.6% of studies did not report injury incidence (general, training, and match); they defined injury differently from injuries/1000 h of exposure; and they did not express the severity, location, and type of injuries as a percentage, nor did they provide sufficient data to allow their calculation. The search process resulted in 46 articles (36 studies on professional football and 10 on amateur football) that met the inclusion criteria. These studies were conducted between 1990 and June 2023 for professional athletes and between 2012 and June 2023 for amateurs. Eligible studies included professional and amateur football players and participants in world tournaments, continental tournaments, and professional leagues from different countries (England, Switzerland, Qatar, Iran, Brazil, USA, Spain, Netherlands, Norway, Italy, Germany, Portugal, Ireland, and Iceland). Figure 1 shows the organizational chart of the study selection process. The supplementary file provides a descriptive summary of the characteristics of the eligible studies. Regarding the quality of study reporting, the mean STROBE quality scale score was 8.55 ± 0.5 (minimum = 5; maximum = 11). Regarding the NOS scale, the mean score obtained was 7.25 ± 0.69 (minimum = 5; maximum = 8). Detailed data for the STROBE and NOS scales are presented in Additional files 6 and 7. No studies were excluded based on the STROBE quality scale and risk of bias assessment.

### 3.2. Incidence of Injuries: General, Training, and Match

In Table 1 and Table 2 are inscribed the values of total incidences at training and at matches for professional men football players [4,11,13,14,16,17,19,23,33,34,35,36,37,38,39,40,41,42,43,44,45,46,47,48,49,50,51,52,53,54,55,56,57,58,59,60,61] and amateur football players, respectively [19,25,26,27,28,29,30,62,63,64,65]. In professional football players, thirty-six studies reported the overall incidence, which varied widely from 1.60, 95% confidence interval, injuries/1000 h [37] and 25.6, 95% confidence interval injuries/1000 h of exposure [38]. The mean overall incidence was 7.75 ± 2.28/lesions/1000 h. Confidence intervals (CI 95%) were not reported in a single study [55], nor could they be calculated from the data reported in the articles. As with total incidence, training incidence was also reported for all 36 eligible studies. The mean value was 3.97 ± 1.35 injuries/1000 h, with a range of variation between 0.8 [37] and 11.8 injuries/1000 h [16]. The mean incidence per match was 30.64 ± 10.28 injuries/1000 h, with limits of variation between 6.16 injuries/1000 h [14] and 97.7 injuries/1000 h [38]. Four other studies reported match incidence values greater than 60, 61.6 [48], 61.86 [52], and 66.0 [40]. For training and match incidences by the same author, confidence intervals were not reported, as for total incidence.

In male amateur football players, 10 studies reported match incidence, three studies did not report total incidence [25,64,65], and 2 studies reported training incidence [25,65]. The mean values were 7.98 ± 2.95; 3.22 ± 1.4, 95% confidence interval, and 17.56 ± 6.15, 95% confidence interval, injuries/1000 h overall incidence, in training and match, respectively.

Correlations between overall, training, and match incidence were established by calculating the Pearson coefficient, which indicates the link between two variables. In the case of professional footballers, the Pearson coefficient values were as follows: 0.675 between overall incidence and training incidence, indicating a moderate association; 0.660 between overall incidence and match incidence, corresponding to a moderate relationship; and 0.304 between training and match incidence—the last value indicates a weak correlation between the two variables. In amateur football players, a weak relationship was found between overall incidence and training incidence (0.556), moderate associations between overall and match incidence (0.603), and 0.822 between training and match incidence, indicating a strong association at a level of statistical significance *p* = 0.05. In all cases the correlations were positive; the increase in the values of one variable was associated with an increase in the values of the other variable.

### 3.3. The Location of the Injuries

*Professional football*. Thirty studies reported lesions location as percentage values, which varied widely by anatomical region and study (Table 3). Injuries to the lower extremities were the most frequent, representing on average 83.32 ± 4.85% of all injuries, with a range of variation between 64.2% [11] and 94% [61]. Injuries to the trunk and upper extremities recorded an average value of 31.1 ± 7.44%. Head/face/neck injuries had the lowest mean value of 3.99 ± 1.8% with a range of 1.0% [16,49] and 12.5% [11], being 21 times less frequent than injuries to the lower extremity. Regarding injuries to the lower extremities, the thigh showed the highest percentage of 26.07 ± 5.53%, followed by the ankle at 15.84 ± 3.93%, the knee at 14.7 ± 2.84% and the hip/groin region at 12.96 ± 2.15%.

**Figure 2:**
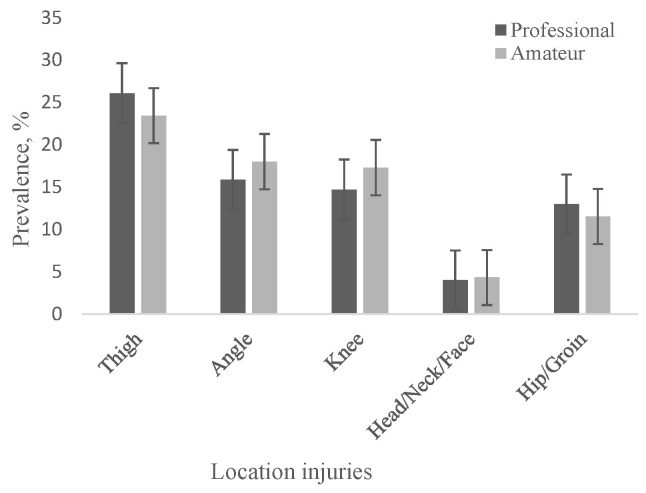
Prevalence of injuries in football by location.

*Amateur football*. The distribution of lesions by anatomical regions in amateur football players was relatively similar to that in professional football players (Table 4). Injuries also predominated in the lower extremity, their average value being 80.4 ± 7.04%, slightly lower than in the case of professional footballers. For head/face/neck injuries, a mean value of 4.32 ± 2.92% was recorded, slightly higher than that found in professional football players. Lower extremity injuries were represented by injuries to the thigh 23.4 ± 7.69%, to the ankle 17.99 ± 3.63, to the knee 17.28 ± 5.26% and in the hip/groin region 11.5 ± 4.97%, values slightly increased compared to those found in professional football players, except for thigh and those in the groin region (Figure 2), where the values were similar. In both cases, the lower leg/Achilles tendon and foot/toe injuries were not recorded separately, they were found in the lower extremity injuries. The relatively small number of studies in amateur football players (7) that reported the distribution of lesions by anatomical region makes it difficult to compare the results with those obtained in professional football players (27 studies).

## 4. Discussions

### 4.1. Risk of Injury

Football is the sport associated with the highest number of injuries as well as the highest injury rate per unit of exposure [66,67]. Injuries occur during football games and practices due to the combination of high speeds and full contact. In our systematic review, we found incidence rates of general, training, and match injuries of 7.75 (5.45–10.03), 3.97 (2.63–5.33), and 30.64 (19.36–40.92) injuries/1000 h of exposure in professional football players (adult men) and 7.89 (4.94–10.84), 3.22 (1.82–4.62), and 17.56 (11.41–23.71)/1000 h in amateur football players, respectively. These values are different from those reported by [22], who found mean values of 10.7, 4.6, and 55.9 injuries/1000 h, for all incidence types (20 cohort studies). Closer incidence rates were obtained by [20], namely 8.01 (7.18–9.00) overall incidence, 3.73 (3.10–4.36) in training, and 36.04 (31.28–40.80)/1000 h per match (group analysis, 46 studies). Palazon et al. [68], in a meta-analysis, indicate injury incidence rates of 6.1, 3.0, and 19.2 injuries per 1000 exposure hours in general, training, and match, respectively, in football-playing females, close to the values reported by [69] in their meta-analysis (5.63; 3.27, and 19.02 injuries/1000 h), lower values than in male professional and amateur football players. Epidemiological data regarding injuries involving male football players cannot be generalized to female football players [70,71] due to anthropometric and physiological differences such as performance and fatigue responses between the sexes [70]. Female players have been found to perform at a slower speed, cover shorter total distances, tire more quickly, and perform less well later in the game compared to male football players [71]. Also, in young footballers, the incidences in training and match of 5.7; 2.8, and 14.4/1000 h, respectively [68], were lower compared to values reported for adult male professional and amateur football players. Compared to other team sports, football has the most injuries. Thus, several studies analyzing the injury profile of handball players found a global incidence of 4.1–12.4 injuries/1000 h of total exposure, an incidence of training injuries between 0.6 and 4.6, and the incidence at a match was between 10.8 and 73.6 injuries/1000 match hours [72,73,74,75], values close to those indicated by us and other authors. As in football in the etiology of injuries in handball, there are several risk factors regarding the number and type of injuries. It was found that the incidence of injury differs according to sex, position in the game, experience, location of the injury, age, category of competition, type of ground, anthropometric variables, and psychological stress. Female handball players had a higher injury incidence rate (6.21/1000 game hours) than male handball players (4.39/1000 game hours), although male players under 18 had an overall risk of 1.76 times more likely to be injured compared to women.

Although basketball is not one of the sports with the highest risk of injury, professional players are the most prone to injury because the games are much more demanding and they have to push their bodies to the limit for long periods. Longo et al. [76] reported 1.3 injuries/1000 h for total incidence and 0.5/1000 and 11.2/1000 exposure hours for practice and match incidence, respectively, significantly lower values compared to data related to men’s and women’s professional and amateur football, adults and youth.

Injuries in volleyball are much less common than in football, basketball, or hockey, but more common than in other non-contact sports such as tennis, skiing, and gymnastics. Unlike contact sports, volleyball injuries are most often the result of a fall, kick, or blocking of the ball during play. Verhagen et al. [77], in a prospective one-season study, showed an overall volleyball injury incidence of 2.6/1000 h of play. The incidence of training-related injuries was 1.8/1000 and for match-related injuries 4.1/1000 h of exposure, values slightly increased from those reported by other authors who indicated an overall injury incidence of 1.7 ± 0.2 per 1000 h of play, 1.5 ± 0.2 during practice, and 3.5 ± 0.8 during match time. In contrast, Bere et al. [73] found the incidence of time-loss injuries during a match to be 3.8/1000 h (95% CI 3.0–4.5); this number is higher for senior players than it is for junior players at 2.04, (1.29–3.21), with no difference between men and women at 1.04, (0.70–1.55)/1000 h.

For adult professional and amateur football players, the incidence rate during a match was higher than during training. Thus, the incidence at a match was 7.8 times higher in our study among professional footballers and 5.4 times higher among amateurs, and 12.2 and 9.7 times higher, respectively, in the case of [20,22] (meta-analyses). Similar trends were found in female football players and youth athletes (6.4 and 5.8 times higher) [68,69].

Several studies have attributed the difference between match and training injury rates to several factors. Stress at live events, fighting mentality and risk-taking behavior, and high expectations from team management and supporters are identified as psychological antecedents of athletic injuries. Stressed players will play robustly and aggressively with the possibility of injuring themselves during a match [78]. Also, the greater physical demands of the game during matches compared to the demands of training sessions, the greater variability and uncertainty in the demands of the game when competing against rivals, the number of contacts and collisions during matches, and the fatigue generated during a match may contribute to a higher prevalence of match injuries relative to training [68].

### 4.2. The Location of the Injuries

In football, the region most exposed to injury is the lower extremity, both in professional male and female football players and in young and adult amateurs. In our systematic review, the prevalence of lower extremity injuries was 83.32% (64.2–94%) in male professional football players and 80.49% (69.8–91.0%) in amateurs. These values are close to the data reported by [79], which indicated, for the lower extremity, injury values between 60 and 91%. Junge et al., [80] reported a lower extremity injury prevalence of 70%, lower than the overall prevalence found by [81] in young and adult English elite footballers: 85%. Lopez et al. [20] found a lower extremity injury incidence rate of 6.8 injuries/1000 h of exposure in adult male football players. Most studies reported upper extremity injuries (not highlighted by us on specific locations, they are found in the tables under other anatomical regions) in second place, followed by head/face/neck injuries. Our group analysis indicates for the head/face/neck area a prevalence of 3.99% (1–12.5%) in professional footballers and 4.32% (0.4–8.0%) in amateur footballers. Fuller et al. [82] reported for head/face/neck injuries an incidence of 3.5 (2.4–4.6) for men and 4.1 (2.1–6.1)/1000 h of exposure for women, in the case of time-wasting injuries. They suggest as risk factors for head/neck injuries in female football players greater peak angular acceleration and neck segment displacement in women than men when heading the ball, lower isometric neck strength, neck circumference, and head mass, which would lead to lower levels of head–neck stiffness. The player actions most likely to cause a head or neck injury consist of using the upper extremity or the head, but in most cases, these challenges are considered to be fair and in accordance with the laws of the game [81].

In our study, the four most common locations of lesions were thigh (26.07 ± 5.53%), ankle (15.84 ± 3.93%), knee (14.7 ± 2.84%), and hip/groin (12.96 ± 2.15%) in professional football players and (23.4 ± 7.69%), (17.99 ± 3.63%), (17.28 ± 5.26%) and (11.5 ± 4.97%), respectively, in amateur football players. Our results differ from the data reported by [79], which indicate the following values: thigh 23%, ankle 17%, knee 17%, and hip/groin 9%, different according to the age of the football players.

The thigh muscles involved in football injuries are usually bi-articular with a complex architecture, with a high percentage of fast-twitch fibers and which undergo eccentric contractions and are capable of producing high forces [83]. The injuries affect four groups of muscles: hamstrings (37%), adductors (23%), quadriceps (19%), and gastrocnemius (13%), with a recurrence rate of 16% and prolonging unavailability by 30% [14]. Previous epidemiological studies have consistently reported that the hamstrings are the most commonly injured muscle group in professional football players. In our review, a relatively small number of studies presented data on thigh muscle group injuries. Thus, Ekstrand et al. [14] reported 12% hamstring injuries, 5% quadriceps, 7% adductors, and 4% calf muscle, noting that these injuries were more numerous during a match than during training.

The biceps femoris (53%) was the most common muscle of the hamstring complex exposed to injury. This finding is supported by other studies. The anatomy of the biceps femoris may help explain its higher injury rate. First, it has a long head and a short head, both with separate nervous systems [84]. This dual innervation can lead to asynchronous stimulation of the two ends [85,86]. The wrong contraction of different parts of the muscle group can mean a reduced ability to generate effective tension to control the loads imposed on the muscle [86].

Knee and ankle injuries are the most common injuries suffered by football players as well as handball, basketball, and tennis players, and alpine skiers. They generate the biggest consequences in terms of absence from the game; the rehabilitation of a knee injury takes an average of 45 days. The group mean values established in our review of 14.7 ± 2.84% in professional football players and 17.28 ± 5.26% in amateur football players are different from those reported by [87], who indicated knee injury values of 14.3% in teenagers and 18.5 in elite athletes participating in tournaments. Many athletes suffer injuries to certain knee structures, such as the anterior cruciate ligament (ACL), posterior cruciate ligament (PCL), medial collateral ligament (MCL), and lateral collateral ligament (LCL), with the ACL being the most commonly affected. Nicolini et al. [88], comparing ACL injuries in different sports, reported 54.5% in football, 47% in handball, 36% in basketball, and 27% in volleyball from knee injuries. The increased risk of ACL injury can be attributed to biomechanical factors at the knee and hip impairments in trunk neuromuscular control [89], and anatomical factors at the hip and knee [90,91]. A female athlete with a low width of the femoral notch and an increased slope of the tibial articular cartilage, oriented posterior-inferior in the lateral compartment and a male athlete with a low volume of the ACL and a low angle of the posterior meniscus of the lateral compartment were most at risk of sustaining an ACL injury [91]. Depending on the gender of the player, the incidence rate of ACL injuries is higher in females, while the incidence rate of ankle sprain is higher in males. Incidence rates of ACL injuries and ankle sprains are higher in players competing at higher levels of play and during games compared to training [92].

Ankle injuries are common in both professional and amateur footballers, especially lateral ligament injuries, as football is a high-impact sport that puts a lot of stress on the ankle. They can be caused by the explosive and shifting movements in football, combined with the constant contact between players. It usually happens when the foot is rolled inward, either from a misstep or from an attack. Some authors reported different values for ankle injuries: 11%; 15%; 22%; 32%; 23% (for both men and women) [93,94,95,96,97], and the results of the National Collegiate Athletic Association indicate 20% for men and 21% for women (adolescents). These values differ from our findings of 15.84 ± 3.93% for professionals and 17.99 ± 3.63% for amateurs. Differences in injury definition and methodology have made comparisons between studies difficult and explain the significant differences in results. Many recent studies have expressed injury as lesions/1000 h of exposure, not as prevalence (percentages), as in our review.

The hip/Groin is a complex anatomical region halfway between the abdomen and the thigh, traversed by a multitude of anatomical structures. The anatomical complexity of the groin area makes hip/groin injuries often a diagnostic challenge for the physician. Groin injuries are common in high-intensity team sports (ice hockey, football, and swimming) [98] and account for 8 to 18% of all football injuries [4,16,41,46,76,98,99,100,101] with an incidence reported to be 0.8 to 1.3 groin injuries per 1000 h of sports activity [16,46,76,99]. Groin injuries appear to be more common among male football players compared to their female counterparts, regardless of injury definition, study design, setting, and level of play. The proportion of groin injuries during club-season play reported was 4–19% (aggregate 12.8%) in men’s football and 2–11% (aggregate 6.9%) in women’s football, suggesting that groin injuries are a greater proportion of the total injury burden in male football players [17,102]. In our review, groin injuries ranged from 3.3–20% for professional footballers and 3.8–14.1% for amateur footballers. The average values obtained of 12.96 ± 2.15% and 11.5 ± 4.97% for the two categories of football players are close to the values reported by the mentioned authors.

### 4.3. Game Level

In our review, several studies identified differences between elite, sub-elite, and amateur adult male players in match and training incidences. Also, the variation of these indicators was noted depending on the country of origin of the football team (climate); type of competition, such as friendly matches or competitive matches (national, world cups, European cups, or the Olympics); season; and lawn type.

In European male professional football, Waldén et al. [102] reported that teams located in northern Europe (England, Belgium, Germany, Holland, Scotland, northern France, and northern Italy), countries that typically have milder summers and longer winters, had a higher incidence of injuries compared to teams from southern Europe (Spain, Portugal, and central or southern Italy) with a Mediterranean climate. Hägglund et al. [16] found that elite players in Denmark had a higher risk of injury during training (11.8 vs. 6.0/1000 h) and a higher rate of injury recurrence than their Swedish counterparts. In addition, the risk of suffering a major injury was more than twice as high in Denmark. Greater training exposure and a longer pre-season period in Sweden (171 vs. 123 h) may explain the reported differences, with Danish players having a more unfavorable training/match exposure ratio than Swedish players. For elite Swedish football teams, Hägglund et al. [17] showed that the incidences of training and match injuries were similar between seasons (5.1 vs. 5.3 injuries/1000 training hours and 25.9 vs. 22.7/1000 match hours). Players injured in the first year had a higher risk of injury the following season compared to uninjured players (hazard ratio 2.7); those with a previous hamstring injury, groin injuries, and knee joint injuries were two to three times more likely to suffer an identical injury the following season, while no such relationship was found for ankle sprains. Age was not associated with an increased risk of injury [17].

Whalan et al. [103] reported an injury incidence rate of 20 injuries/1000 h of exposure and an injury burden of 228 days/1000 h of play in Australian sub-elite footballers. Muscle and ligament injuries were most prevalent (41% and 26%) and carried the highest injury burden (83 and 80 lost days/1000 h, respectively). The match incidence values of the Swedish elite footballers were higher than the Australian sub-elite footballers, and the injury severity profile was also relatively different. The same finding was reported by [68], who indicated a match injury incidence in elite male players of 17.9 injuries/1000 h, with 7.3 injuries/1000 h higher than the incidence of match injuries in sub-elite male football players (10.6 injuries/1000 h). In European professional leagues, there is considerable variation in the number of matches played per season (some clubs have had 60–76 team matches). Higher-level players are thus forced to play more matches, especially in the final period of the season, which leads to fatigue and increases the risk of injury.

Between South American teams and European teams, Bengtsson et al. [104] found no significant differences in injury incidence. Higher proportions of training exposure make the incidence of training injuries higher, with 59% of all injuries reported in the South American cohort occurring in training. In contrast, in the European cohort, the majority of injuries (58%) occurred during a match.

Asian professional football is characterized by an overall injury incidence similar to that reported in Europe but with a high rate of ACL tears and hamstring injuries (54.4%) [15]. Incidences of injuries during a match of 14.5 and 19.2 injuries/1000 h, respectively, were significantly higher than in training (2.8 and 4.4/1000 h) [15,105], but injury patterns showed no major differences. In addition to normal league matches, successful teams often participate in national and international cups. It is believed that too many matches can lead to a lack of motivation and mental exhaustion, as players are no longer able to adapt themselves for matches and training. Impaired concentration can affect coordination and lead to underperformance and a greater risk of injury. It is believed that the major stressors are not the 90 min of the match itself, but the mental preparation for matches, travel, and possibly adapting to changes in weather and climate.

The incidence and characteristics of football injuries during matches in high-level international tournaments such as the Olympic Games, European Championships, and World Cups are well documented [15,46,48,61,106,107]. The incidence of time-loss injuries in matches was 50.7 injuries per 1000 h at the 2002 FIFA World Cup [107], 45.9 injuries/1000 h at the 2006 FIFA World Cup [106], 36.0 at EURO 2004 (Waldén et al., 2007), 41.6 at EURO 2008 [46] and 2.4 injuries/1000 h at the FIFA World Cup 2010 [39]. The incidence of training injuries at the European Championships ranged from 0 to 3.9 time-losing injuries per 1000 h of training [46,61].

Several studies have reported injury characteristics during playing on artificial turf compared to playing on natural grass in both male and female elite players [108,109,110,111], young players [112], and amateurs [113,114], as well as during tournaments [115]. The overall risk of injury did not show major differences between the two types of surfaces. However, there were differences in the pattern of injuries sustained. On pitches with new-generation artificial turf, the risk of muscle strain was lower, but the risk of sprained ankles was higher.

Compared to professional footballers, the incidence of injuries during training was higher among amateur footballers (3.9 vs. 2.1/1000 h), in contrast to the incidence of injuries among professionals, which was higher during matches (31.8 vs. 20.4 injuries/1000 h of exposure) [27]. These values are different from those reported by [19] for top-level (25.3 and 3.8 for match and training incidence), elite (23.5 and 4.9), and amateur players (12.3 and 2.7 injuries/1000 h). Our aggregated results are also relatively different compared to those indicated by [19,27]. The match incidence in professional footballers was higher than in amateurs (30.64 vs17.56/1000 h), but similar to that reported by [27] at 31.8/1000 h, and higher than the values indicated by [19] for top-level and elite players. Incidences in training were similar (3.97 in professionals and 3.22/1000 in amateurs), but 1.88 injuries/1000 h higher in professional football players (3.98 vs. 2.1) [27], with 0.32 and 1.72 injuries/1000 h lower than top-level and elite, respectively, but higher with 0.52 injuries/1000 h compared to amateur football players [19]. The intensity of training sessions and matches increases with the level of competition, so skilled players are at greater risk of injury than less skilled players. Professional players had a lower incidence of moderate and severe injuries than amateur players, but a higher incidence of minimal injuries. These differences can be explained by the smaller number of players in amateur teams, with fewer options to replace injured or injury-prone players. Thus, amateur players had a higher match exposure per player than professionals by 17% [19]. In the case of minor injuries in the amateur cohort, there is the possibility of their underreporting due to the reduced contact between medical staff and players (2–3 times a week, during training and matches), which contrasts with the daily contact between medical staff and players in professional football. As a result, some minor amateur injuries may not be recorded due to players recovering from minor injuries in the meantime. Also, medical support is less consistent given the economic constraints of amateur teams, leading to delayed diagnoses and suboptimal rehabilitation, incomplete wound healing, and/or neglect of minor injuries [46,116].

### 4.4. Limitations

Our study, conducted according to international guidelines for systematic reviews, has some limitations. They are related, in part, to the limited availability of some databases and the use of only articles published in English, which do not exclude the possibility that some valuable studies were not selected by us. The lack of uniform data collection methods can be sources of inconsistency in results, as can differences between national leagues in terms of climatic regions, peak match periods, number of matches and breaks, and level of professionalism. Also, the large variation in sample sizes, the limited geographical areas of the studies (mostly in Europe, especially in Sweden, Spain, Holland, and Germany; the results cannot be generalized to other regions), and the different durations of investigation (from several weeks, at one season, and two or more seasons) could partially explain the heterogeneous estimates obtained in our systematic review. Furthermore, it is not excluded that some studies may have reported not only “time loss” injuries, but all injuries, without specifying this. This discrepancy in injury definitions between studies would partially account for the different incidences of injuries observed. We did not include in the analysis injuries that required medical attention, which might have led to higher incidence rates, nor was it possible to report injury burden due to a lack of data on the number of days lost per injury in some of the studies included. In both professional and amateur footballers, our data extraction template allowed the documentation of only the data related to the location of the injury and the type and severity of injuries by main groups and not stratified by subgroups, also motivated by the fact that not all studies eligible showed a complex stratification. We did not allow space for interactive effects within risk factors such as pitch type (especially in amateur footballers), playing position, assessment of protective equipment used by players, violation of competition rules, and quality of referees. Exposure of amateur athletes to additional interventions outside of game protocols as part of their normal routine is not excluded, but their monitoring was not recorded or feasible in some studies. The small number of eligible studies in amateur football players did not allow us to make a satisfactory comparison with professional football players. The life of a professional footballer is totally different from the life of an amateur player. Professional football players benefit from customized training programs with better equipment and a variety of training programs. Retrospective study designs may have increased bias in assessing accurate injury history.

## 5. Conclusions

The main findings of this study were as follows: the overall injury incidence rate was higher in amateur compared to professional footballers, but the training and match incidence rates were higher in professionals than in amateurs. For both categories of footballers, the incidence at a match was more than five times higher than the incidence at training, the highest values being observed during the European and world championships. Lower extremity injuries, thigh, ankle, and knee injuries predominated. Professional and amateur football players remain athletes at substantial risk of injury, especially during matches that require the highest level of performance. The current findings have implications for the management, monitoring, and design of training, competition, injury prevention (especially severe injuries), and education programs for amateur football players.

## Figures and Tables

**Table 1:** Incidence of injuries and confidence interval: overall, training, and match (Professional football).

Studies Professional Football	Overall Incidence/ 1000 h	Confidence Interval CI 95%	Training Incidence/ 1000 h	Confidence Interval CI 95%	Match Incidence/ 1000 h	Confidence Interval CI 95%
Arnason et al., 2005 [35]:						
Intervention Group	6.6	5.41–8.05	2.7	1.91–3.81	25.1	19.61–32.13
Control Group	6.6	5.41–8.04	1.9	1.25–2.88	26.0	20.17–32.63
Aus de Fünten et al., 2014 [36]:						
Season 2008–2009	5.90	5.03–6.82	2.67	2.08–3.44	31.5	25.0–38.0
Season 2009–2010	6.55	5.58–7.69	3.98	3.19–4.95	26.5	20.2–32.7
Aus der Fünten et al., 2023 [23]	5.5	5.3–5.6	3.4	3.3–3.6	25.9	25.0–26.9
Bayne et al., 2018 [37]	1.6	1.05–2.15	0.8	0.40–1.20	16.5	8.87–24.13
Dupont et al., 2010 [38]:						
Group 1	4.1	3.0–5.1	2.5	1.6–3.4	19.3	11.9–26.7
Group 2	25.6	20.8–30.5	8.3	5.2–11.4	97.7	76.1–119.2
Dvorak et al., 2011 [39]	9.20	7.68–10.72	4.40	3.27–5.53	40.10	31.42–48.78
Eirale et al., 2010 [40]	7.8	6.24–9.75 *	4.3	4.06–4.56 *	66.0	47.83–91.08 *
Eirale et al., 2013 [41]	6.0	4.90–6.50	4.4	3.7–5.2	14.5	11.6–18.0
Ekstrand et al., 1990 [42]:						
Division I	8.6	7.56–9.64	4.6	3.73–5.47	21.8	18.42–25.18
Division II	8.5	7.51–9.49	5.1	4.21–5.99	18.7	15.55–21.85
10. Ekstrand et al., 2004a [43]:						
Europe: Non world Cup	9.5	9.5–12.8	5.5	5.6–8.5	30.3	26.5–38.2
Europe: World Cup	7.9	7.7–11.6	3.2	2.3–4.5	26.7	26.7–46.8
11. Ekstrand et al., 2004b [44]	10.0	7.3–12.7	6.5	4.1–8.9	30.3	18.4–42.1
World Cup	-	-	-	-	43.3	15.9–70.6
Non world Cup	-	-	-	-	28.5	15.4–41.6
12. Ekstrand et al., 2011a [14]	8.0	4.6–11.4 *	4.1	2.1–6.1 *	27.5	16.7–38.3 *
13. Ekstrand et al., 2011b [33]:						
SWE	2.58	2.38–2.79	1.67	1.50–1.86	8.75	7.74–9.88
UEFA UCL	2.70	2.58–2.83	1.38	1.29–1.48	9.58	9.02–10.48
ART	1.82	1.67–1.99	1.11	0.98–1.25	6.16	5.42–7.00
14. Häggland et al., 2005a [16]:						
Denmark	14.4	9.1–19.8	11.8	6.7–16.9	28.2	17.8–38.7
Sweden	8.2	5.5–11.0	6.0	3.9–8.2	26.2	16.8–35.5
15. Häggland et al., 2006 [17]:						
Sweden; Seasons 2001	7.6 *	7.1–8.3 *	5.1 *	4.6–5.6 *	25.9 *	22.8–29.2 *
Sweden; Seasons 2002	7.6 *	7.0–8.3 *	5.3 *	4.7–5.8 *	22.7 *	20.0–25.8 *
16. Häggland. 2007 [45]:						
Season 1982	8.3	5.1–11.8	4.6	2.7–6.6	20.6	14.0–27.1
Season 2001	7.8	5.2–10.4	5.2	3.4–7.1	25.9	16.7–35.2
Season 2002	7.6	7.0–8.3	5.3	4.7–5.8	22.7	20.0–25.8
Season 2005	7.7	7.1–8.3	4.7	4.2–5.3	28.1	24.8–31.8
Hägglund et al., 2009 [46]:						
Men’s Under-21 2006 European Championship	13.8	9.1–21.0	4.6	1.9–11.2	33.1	20.6–53.3
Men EURO 2008. European Championship	10.4	8.0–13.6	2.8	1.6–4.9	41.6	30.9–55.9
Men’s Under-21 2007 European Championship	10.8	7.3–15.9	3.4	1.5–7.5	34.7	22.1–54.4
Hägglund et al., 2013 [47]	7.7	1.7–20.5	4.0	0.5–12.1	26.6	6.7–57.0
Häggland et al., 2016 [19]:						
Top level European footballers	7.2	7.0–7.3	3.8	3.7–3.9	25.3	24.7–25.9
Elites	7.4	7.1–7.6	4.9	4.7–5.1	23.5	22.4–24.6
Hawking et al., 1999 [13]	8.5	7.81–9.19	3.4	2.91–3.89	25.9	23.33–28.47
Jones et al., 2019 [11]	9.11	6.48–12.87	6.84	3.98–9.70	24.29	19.92–28.65
Lee et al., 2014 [48]	7.4	6.61–8.29 *	3.4	2.86–4.05 *	61.6	52.67–70.88 *
Mallo et al., 2011 [49]	10.9	8.7–13.0	5.2	3.9–6.6	44.1	33.5–54.6
Martins et al., 2022 [50]:						
Season 2019/2020	3.3 *	2.24–4.85 *	2.4	1.49–3.86 *	14.0	7.29–26.86 *
Season 2020/2021	4.4 *	3.15–6.14 *	2.8	1.81–4.34 *	21.9	13.04–36.79 *
Season 2021/2022	3.1 *	2.08–4.62 *	2.2	1.37–3.54 *	10.8	5.14–22.68 *
Morgan et al., 2001 [51]	6.2	-	2.9	-	35.3	-
Murphy et al., 2012 [52]	8.89 *	8.35–9.47 *	4.05	3.67–4.47	61.86	56.91–67.23
Noya et al., 2014b [53]. Second Division	5.51	5.15–5.87	3.77	3.46–4.08	38.83	34.53–43.14
Noya et al., 2014a [54]; Premier league Spain	5.65	5.35–5.96	3.55	3.31–3.81	43.53	39.96–47.42
Parry Drust et al., 2001 [55]	6.2		1.8		24.6	
Reis et al., 2015 [56]	5.37 *	4.25–6.69 *	2.40	1.62–3.18	42.84	29.73–55.95
Roe et al., 2018 [57]	8.5 *	8.07–8.95 *	3.85	3.56–4.17	49.8	46.85–52.94
Shalaj et al., 2016 [58]	7.38	7.14–7.63	3.16	2.70–3.69	35.37	31.96–39.14
Stubbe et al., 2015 [59]	6.2	5.5–7.0	2.8	2.3–3.3	32.8	28.2–38.1
Waldén et al., 2005a [60]	9.4	7.3–11.5	5.8	3.6–6.4	30.5	23.1–37.9
Waldén et al., 2005b [4]:	7.66 *	7.13–8.23 *	5.15 *	4.68–5.62 *	25.45 *	22.7–28.5 *
Waldén et al., 2007 [61]:						
Europa 2004	10.1	7.24–12.96	2.10	0.62–3.58	36.0	24.51–49.49
U-19 EC 2005	13.40 *	7.37–19.48	2.92	0.62–8.88	30.4	13.42–47.38
Average Values ± SD	7.75 ± 2.28		3.97 ± 1.35		30.64 ± 10.28	

* Calculated values. SWE—Swedish Super League; UEFA UCL—Champions League; ART—artificial turf field.

**Table 2:** Incidence of injuries and confidence interval: overall, training, and match (amateur football).

Studies Amateur Football	Overall Incidence/ 1000 h	Confidence Interval CI 95%	Training Incidence/ 1000 h	Confidence Interval CI 95%	Match Incidence/1000 h	Confidence Interval CI 95%
Brito et al., 2012 [25], Total	8.6	7.48–9.88	6.5	5.52–7.65	34.9	27.0–45.0
U-19	11.4	9.12–14.25	-	-	-	-
Ficsher et al., 2017 [62]	4.05	3.11–5.19	2.05	1.33–3.02	11.96	8.42–16.48
Gebert et al., 2018 [29]: 2004						
League 2–3	-	-	2.6	2.1–3.2	16.0	13.9–18.7
League 4–5	-	-	2.7	2.2–3.2	17.1	15.1–19.0
League 30+/40+	-	-	4.9	3.6–6.3	22.7	19.7–25.6
Man’s 16–20 years	-	-	2.3	1.8–2.7	12.2	10.4–13.9
Gebert et al., 2018: 2008 [29]						
League 2–3	-	-	2.1	1.6–2.6	14.0	11.8–16.2
League 4–5	-	-	3.1	2.6–3.7	16.5	14.4–18.6
League30+/40+	-	-	6.0	4.3–7.7	18.0	14.9–21.1
Man’s 16–20Years	-	-	1.9	1.5–2.3	12.1	10.5–13.8
Gebert et al., 2018 [29]: 2015						
League 2–3	-	-	2.8	2.2–4.3	17.2	14.5–19.9
League 4–5	-	-	3.5	2.9–4.1	19.0	16.8–21.3
League 30+/40+	-	-	5.0	3.6–6.4	24.2	20.5–27.8
Man’s 16–20 years; 2015	-	-	2.7	2.2–3.2	14.4	12.4–16.4
Häggland et al., 2016 [19]	5.2	4.4–6.1	2.7	2.1–3.6	12.3	9.9–15.3
Hammes et al., 2014 [63]:						
Group Intervention	12.2	8.9–15.6	5.5	2.8–8.1	28.3	18.9–37.6
Group Control	12.6	8.5–16.7	8.1	4.0–12.1	20.5	11.9–29.1
Herrero et al., 2014 [64]:						
Overall	-	-	0.49	0.48–0.5	1.15	1.12–1.18
<30 years	-	-	0.9	-	1.3	-
>30 years	-	-	0.4	-	0.6	-
Kekelekis et al., 2023 [30]	5.5	4.45–6.09	3.077	2.16–3.80	20.76	15.28–26.24
Kordi et al., 2011 [65]:						
ATF	-	-	-	-	19.5	12.9–28.5
DF	-	-	-	-	36.9	28.7–46.6
Nogueira et al., 2017 [28]: U-19	4.02	2.53–5.52	1.97	0.94–3.01	16.01	9.32–22.69
Sousa et al., 2013 [26]	4.86	4.36–6.37	2.39	1.94–2.94	22.75	19.11–27.05
van Beijsterveldt et al., 2012 [27]:						
Group Intervention	9.6	8.4–11.0	3.7	2.8–4.8	21.1	17.8–25.0
Group Control	9.7	8.5–11.1	3.1	2304.1	22.7	19.3–26.7
Average values ± SD	7.98 ± 2.95		3.22 ± 1.4		17.56 ± 6.15	

ATF—artificial turf field; DF—dirt field.

**Table 3:** Location of injuries in professional football players.

Study Professional Football	Lower Extremity,%	Thigh,%	Ankle,%	Knee,%	Head/Neck/Face,%	Hip/Groin,%	Other Anatomical Regions,%,
Arnason et al., 2005 [35]:							
Intervention	80.87	27.7	13.8	18.1	4.26	8.46	27.68
Control	82.3	31.2	16.7	12.5	7.3	8.3	24.0
Aus de Fünten et al., 2014 [36]:							
Season 2008–2009	86.1	33.1	17.2	16.6	2.6	3.3	27.2
Season 2009–201	79.19	25.5	16.11	24.83	6.04	4.7	22.82
Aus der Fünten et al., 2023 [23]	84.5	23.6	12.9 *	15.4	4.29 *	16.84 *	26.97
Bayne et al., 2018 [37]	87.8 *	6.1 *	42.4 *	15.2 *	6.1 *	9.1 *	21.1 *
Dupont et al., 2014 [38]							
Group G1	89.66 *	25.86 *	10.34 *	10.34 *	5.17 *	17.25 *	31.04 *
Group G2	88.80 *	29.9 *	20.6 *	14.08	1.9 *	12.1 *	21.5 *
Dvorak et al., 2011 [39]	83.1 *	19.8 *	15.4 *	15.4 *	5.6 *	4.9 *	38.9 *
Eirale et al., 2010 [40]	73.1 *	33.3	16.7	9.0	3.9	1.3	35.8
Eirale et al., 2013 [41]	-	39.2	12.0	15.2	-	-	33.6
Ekstrand et al., 2011a [14]	87.0	23.8	18.3	14.0	2.3	13.7	27.9
Hägglund et al., 2005a [16]							
Denmark	89.0 *	22.0	21.0	13.0	1.0	15.0	28.0
Sweden	86.0 *	23.0	15.0	11.0	2.0	16.0	33.0
Hägglund et al., 2006 [17]:							
Season 2001	87.68 *	22.96 *	9.65 *	15.31 *	2.67 *	15.97 *	33.44 *
Season 2002	86.56 *	22.28 *	9.35 *	18.37 *	3.06 *	18.71 *	28.23 *
Hägglund et al. 2009 [46]:							
Men Under-21, 2006	-	29	16	16	2	20	17
Men Under -21, 2007	-	20	16	20	8	11	25
Men EURO, 2008	-	21	16	19	7	13	24
Hammes et al., 2014 [63]:							
Group Intervention	84	30.0	-	12.0	-	-	58.0
Group Control	81	33.0	-	11.0	-	-	56.0
Hawking et al., 1999 [13]	87.18 *	22.8 *	16.8 *	14.9 *	2.42 *	13.3 *	29.88 *
Jones et al., 2019 [11]	64.2	31.7	13.0	14.6	12.5	-	28.2
Lee et al., 2014 [48]	83.0 *	14.9 *	11.8 *	16.2 *	2.4*	11.8 *	42.9 *
Mallo et al., 2011 [49]	89	35.0	10.0	14.0	1.0	17.0	23.0
Morgan et al., 2001 [51]	77.0	-	21.1	18.0	-	-	60.9
Noya et al. et al., 2014b [53]	86.9	31.6	11.9	12.8	3.3	16,3	24.1
Murphy et al., 2012 [52]	76.0	33.3	11.3	10.0	3.6	12.5	29.3
Noya et al., 2014a [54]	89.6	36.6	14.3	11.4	2.7	13.5	21.5
Roe et al., 2018 [57]	70.9	33.2	11.7	11.1	3.1	14,9	26.0
Shalaj et al., 2016 [58]	72.0	16.2	22.1	9.6	2.9	8.1	41.1
Stubbe et. al., 2015 [59]	82.9	23.1	10.5	21.32	3.84	10.5	30.74
Waldén et al., 2005a [60]	85.5	23.1 *	13.53 *	19.9 *	3.34 *	12.01 *	28.11
Waldén et al., 2005b [4]	87.0	23.0	10.0	16.0	2.0	16.0	33.0
Waldén et al., 2007 [61]							
EURO 2004	84.4	22.2 *	15.6 *	11.1 *	-	11.1 *	40.0 *
U-19 2005	94.0	23.5 *	29.4 *	11.8 *	5.5 *	11.8 *	18.0 *
Average value ± SD **	83.32 ± 4.85	26.07 ± 5.53	15.84 ± 3.93	14.7 ± 2.84	3.99 ± 1.8	12.96 ± 2.15	31.1 ± 7.44

* Calculated values; ** Mean values and standard deviations were calculated considering only studies that reported values for that parameter; SD—Standard deviation.

**Table 4:** Location of injuries in amateur football players.

Study Amateur Football	Lower Extremity,%	Thigh,%	Ankle,%	Knee,%	Head/Neck/Face,%	Hip/Groin,%	Other Anatomical Regions,%.
Brito et al., 2012 [25], U-19	87.0 *	30.0	18.0	12.0	2.0	7.0	11.0
Kekelikis et al., 2023 [30]	91.0	36.9 *	12.6 *	10.7 *	-	26.2 *	13.6 *
Kordi et al., 2011 [65]:							
ATF	85.1 *	14.8	25.9	18.5	7.4	-	33.4
DF	72.9 *	15.7	14.3	24.3	5.7	-	40.0
Hammes et al., 2014 [63]	84.0	33.0	-	12.0	-	-	55
Herrero et al., 2013 [64]	69.8	10.4	12.4	29.9	8.6	3.8	34.9
Nogueira et al., 2017 [28] U-17 + U-19	85.5	24.6	21.4	13.7	0.4	14.1	25.8
Sousa et al., 2013 [26]	85.0	22.1	19.2	20.2	1.8	8.0	28.7
Van Beijsterveldt et al., 2012 [27]:							
Group Intervention	69.9	-	21.8	11.7	-	9.7	56.8
Group Control	73.8	-	16.3	19.8	-	11.4	52.5
Average value ± SD **	80.4 ± 7.04	23.4 ± 7.69	17.99 ± 3.63	17.28 ± 5.26	4.32 ± 2.92	11.5 ± 4.97	35.17 ± 12.7

* Calculated values; ** Mean values and standard deviations were calculated considering only studies that reported values for that parameter; SD—Standard deviation.

## Data Availability

Data are contained within the article.

## Supplementary Materials

The following supporting information can be downloaded at: https://www.mdpi.com/article/10.3390/jcm12175569/s1. Supplementary Table S1 PRISMA checklist [117]; Supplementary Table S2 Definitions used to include studies in systematic review [118]; Supplementary Table S3. Characteristics of the studies included in the review: General descriptors of study; Description of the study population (*n* = 48); Supplementary Table S4 Characteristics of the studies included in the review: Epidemiological descriptors Methodological quality; Supplementary Table S5 Analysis of the selected studies’ methodological quality - STROBE (*n* = 46) [119]; Supplementary Table S6 Risk of bias assessment of the studies (Newcastle-Ottawa scale; *n* = 46).
